# Rapid isotopic exchange in mineralogically unaltered coral skeletons

**DOI:** 10.1038/s41598-025-06327-9

**Published:** 2025-07-01

**Authors:** Jarosław Stolarski, Deyanira Cisneros-Lazaro, Arthur Adams, Katarzyna Janiszewska, Anders Meibom

**Affiliations:** 1 Institute of Paleobiology, Polish Academy of Sciences, 00-818 Warsaw, Poland; 2 Laboratory for Biological Geochemistry, School of Architecture, Civil and Environmental Engineering, Ecole Polytechnique Fédérale de Lausanne (EPFL), 1015 Lausanne, Switzerland; 3 Center for Advanced Surface Analysis, Institute of Earth Science, University of Lausanne, 1015 Lausanne, Switzerland

**Keywords:** Geochemistry, Palaeontology

## Abstract

Fossil coral skeletons that retain their original aragonitic mineralogy are generally considered diagenetically unaltered and reliable paleoenvironmental archives. We investigated whether oxygen isotope exchange can occur between these skeletons and ambient sediment pore water without aragonite-to-calcite transformation, focusing on the role of skeletal microstructure in this process. Simulated diagenesis experiments using modern coral skeletons immersed in ^18^O-enriched artificial seawater revealed that microstructural complexity – specifically, the phylogenetically controlled arrangement of rapid accretion and thickening deposits – strongly influences isotopic exchange. Organic-rich regions within the skeleton facilitate water penetration, leading to heterogeneous isotopic alteration correlated with skeletal architecture. This provides a mechanism to modify primary isotopic compositions without obvious signs of diagenetic alteration, challenging the assumption that a preserved carbonate polymorph ensures isotopic fidelity in paleoenvironmental reconstructions and complicating the interpretation of the oxygen isotope paleo-temperature proxy in fossil aragonitic biocarbonates. However, we also observed that coral skeletons with less ultrastructural complexity exhibited lower susceptibility to isotope exchange, suggesting a criterium to identify the fossil specimens most suitable for paleoenvironmental analysis.

**Supplementary Information:**

The online version contains supplementary material available at 10.1038/s41598-025-06327-9.

## Introduction

**Fig. 1:**
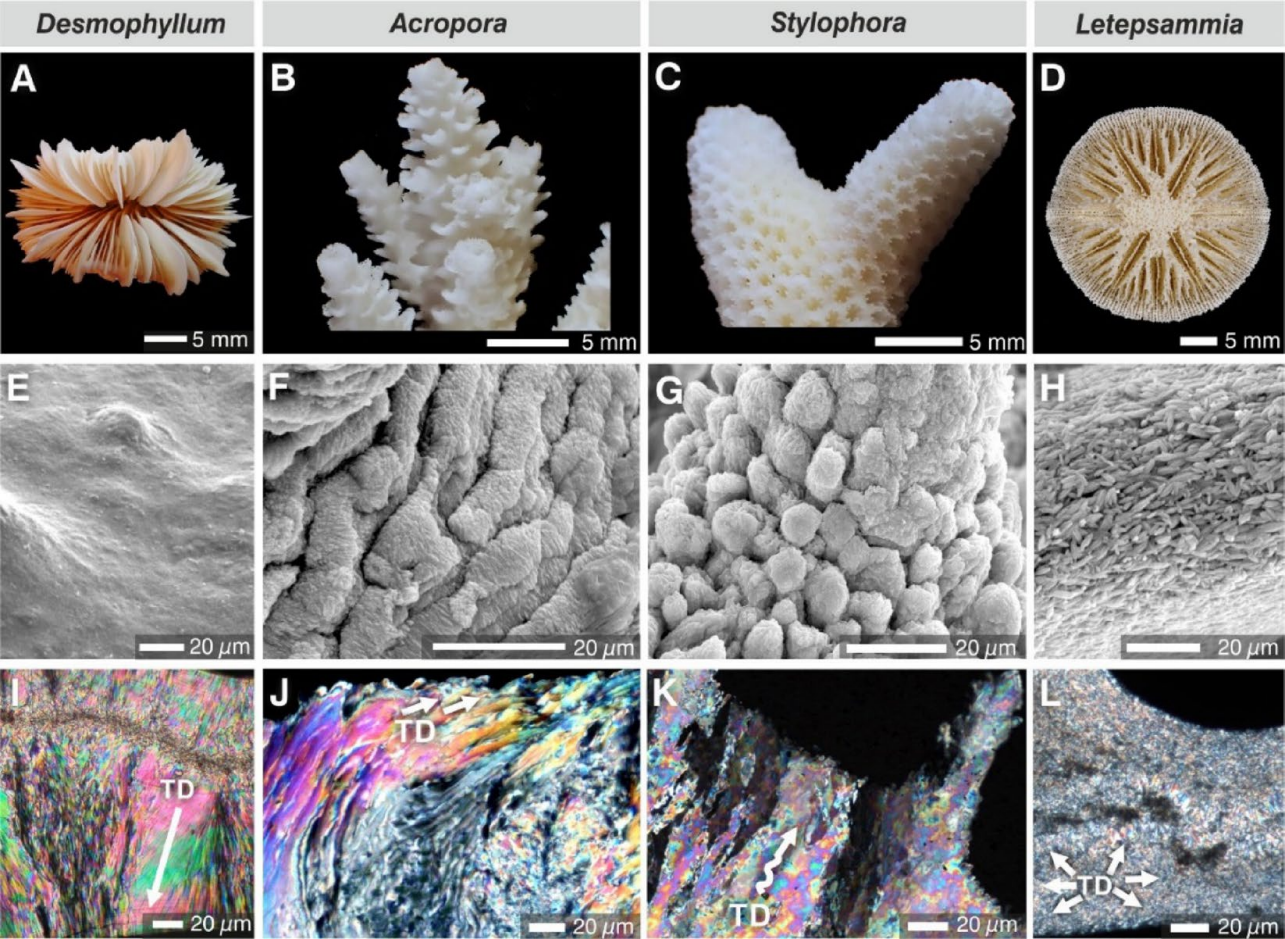
Four scleractinian corals with fundamentally different skeletal architectures subjected to isotopic exchange experiments. (**A**–**D**), Macromorphology (optical photography), (**E**–**H**, skeletal surface textures (SEM), and (**I**–**L**), corresponding microstructural organization of skeleton in transverse sections (optical microscopy, polarized light): TDs in *Desmophyllum* (**I**) consists of crystallographically well-aligned bundles of fibers (arrow) oriented perpendicular to the skeleton surface; in *Acropora* (**J**) elongated packages of TDs bundles are semi-parallel to skeleton surface; in *Stylophora* (**K**) fibers show patchy colors due to quasi-aligned distribution of fibers (wavy arrow); in *Letepsammia* (**L**) chaotically arranged fiber bundles cause poor light extinction within the groups of fibers (arrows).

Fossil skeletons originally composed of metastable calcium carbonate (CaCO₃) polymorphs, such as vaterite, high-magnesium calcite, or aragonite, are considered exceptionally well-preserved if their original mineralogy is preserved. A prominent example is the skeletons of scleractinian corals whose aragonitic coralla, often recovered from impermeable clay deposits, are present in the fossil record across at least 240 My of geological time^1^. These pristine specimens serve as valuable archives of trace element and isotopic compositions that can be interpreted for reconstruction of past seawater chemistry and temperatures^2,3^. In such exceptionally well-preserved coral skeletons, the two primary microstructural components can usually still be distinguished, i.e., rapid accretion deposits (RADs; aka ‘centers of calcification’), which typically make up around 3–5% of the skeletal volume, and thickening deposits (TDs; aka ‘fibers’) that constitute the bulk of the skeleton^4^. Due to their compact crystalline structure, TDs are traditionally considered less susceptible to diagenesis, whereas RADs, characterized by a microcrystalline structure and higher enrichment in organic components, are considered more vulnerable to such alteration^4,5^. However, this perception has been challenged by studies revealing higher structural complexity than previously assumed, with distinct coral groups displaying fundamentally different TD organization^6,7^. These findings suggest that TDs in different coral species may vary in their susceptibility to diagenesis. Furthermore, recent experiments, in which both fossil and modern calcitic tests of benthic foraminifera underwent extensive oxygen isotope exchange with pore water analogues, without modification of structural features^8–11^, hint at a potential hard-to-detect bias in paleoenvironmental reconstructions. This susceptibility to alteration is thought to stem from the nanocomposite structure of the foraminifera tests, which in fact is a key feature of almost all marine biogenic carbonates, scleractinian corals included. This structure, which is likely to be the result of non-classical crystallization processes involving an amorphous calcium carbonate precursor and a range of organic compounds^12–15^permits rapid fluid penetration and provides reactive surface areas orders of magnitude greater than those of abiotic minerals^9–11^. Consequently, this study addresses two fundamental questions regarding the use of coral skeletons in paleoenvironmental research: Can substantial oxygen isotope exchange be experimentally induced in coral skeletons exposed to seawater analogues without a phase transformation from aragonite to calcite? If yes, do different TD microstructural patterns influence this isotopic exchange? To answer these questions, we performed diagenetic experiments using four microstructurally distinct skeletons of modern scleractinian corals (Fig. 1A-D).

## Materials and methods

Scleractinian coral taxa selected for this study (all housed at the Institute of Paleobiology, Polish Academy of Sciences, Warsaw (ZPAL) represent phylogenetically distinct groups: solitary *Desmophyllum dianthus* represents robust clade family Caryophylliidae [ZPAL H.25/5], colonial *Acropora hyacinthus* represents complex clade family Acroporiidae [ZPAL H.25/195(536 A)], colonial *Stylophora pistillata* represents robust clade family Pocilloporidae [ZPAL H.25/219(636)], whereas solitary *Letepsammia formosissima* represents basal clade family Micrabaciidae [ZPAL H.25/8]^6,16–18^. For simplicity, only the generic names (i.e., *Desmophyllum*, *Acropora*, *Stylophora*, and *Letepsammia*) were used in the main text of the paper, with each reference pertaining to the species described above. Small skeletal fragments (approximately 5 mm in length, taken from septa) were broken into smaller pieces such that they could fit into gold capsules measuring 5 mm in diameter, to which seawater analogue was added and the gold capsule subsequently sealed by welding. Samples intended for NanoSIMS imaging were incubated for 7 days at 50 °C in the ^18^O-enriched seawater analogue with an ^18^O/^16^O ratio of 0.30 (i.e., highly enriched in ^18^O) and saturated with respect to aragonite. For bulk measurements, the samples were exposed for 7 days at 50 °C to ^18^O-enriched seawater analogue with an ^18^O enrichment of δ^18^O_VSMOW_ = 1000‰, i.e., the experimental conditions were slightly modified compared with those established for similar work on calcitic foraminifera tests^9,10^.

Subsequent analytical work included scanning electron microscopy (SEM) observations of the skeletal fragments before (control) and after incubation, extensive Raman spectroscopy and mapping to determine the carbonate polymorph, measurements of oxygen isotope composition using a Finnigan Delta V Advantage mass spectrometer coupled to a GasBench II, and NanoSIMS imaging to examine the resulting distribution of ^18^O-enrichment.

Details of isotopic exchange experiments, Raman analyses, bulk and NanoSIMS isotope analyses, and structural SEM observations are provided in the Supplementary Information.

## Results and discussion

Our experiment aimed to test the hypothesis that surface texture (Fig. 1E–H), which reflects the underlying skeletal microstructure (Fig. 1I–L), is a key factor influencing water penetration and the isotopic exchange of oxygen (and other elements). For example, the skeleton of *Desmophyllum* has a relatively smooth surface that reflects an internal arrangement of crystallographically well-aligned bundles of fibers perpendicular to the skeleton surface (Fig. 1E, I). In contrast, the surface texture of *Acropora* skeletons is a shingle-like pattern (Fig. 1F), created by elongated packages of TDs bundles of fibers that make up the bulk of the skeleton (Fig. 1J). The skeletal surface texture of *Stylophora* consists of small tubercles (Fig. 1G). Cross-sections reveal that bundles of TDs fibers exhibit a quasi-aligned distribution (seen as patchy colors),  although their overall orientation is perpendicular to the skeletal surface (Fig. 1K). The skeleton of *Letepsammia* is relatively smooth, composed of small, randomly oriented fiber bundles (Fig. 1H). Cross-sections also reveal randomly arranged fiber bundles (Fig. 1L).

**Fig. 2:**
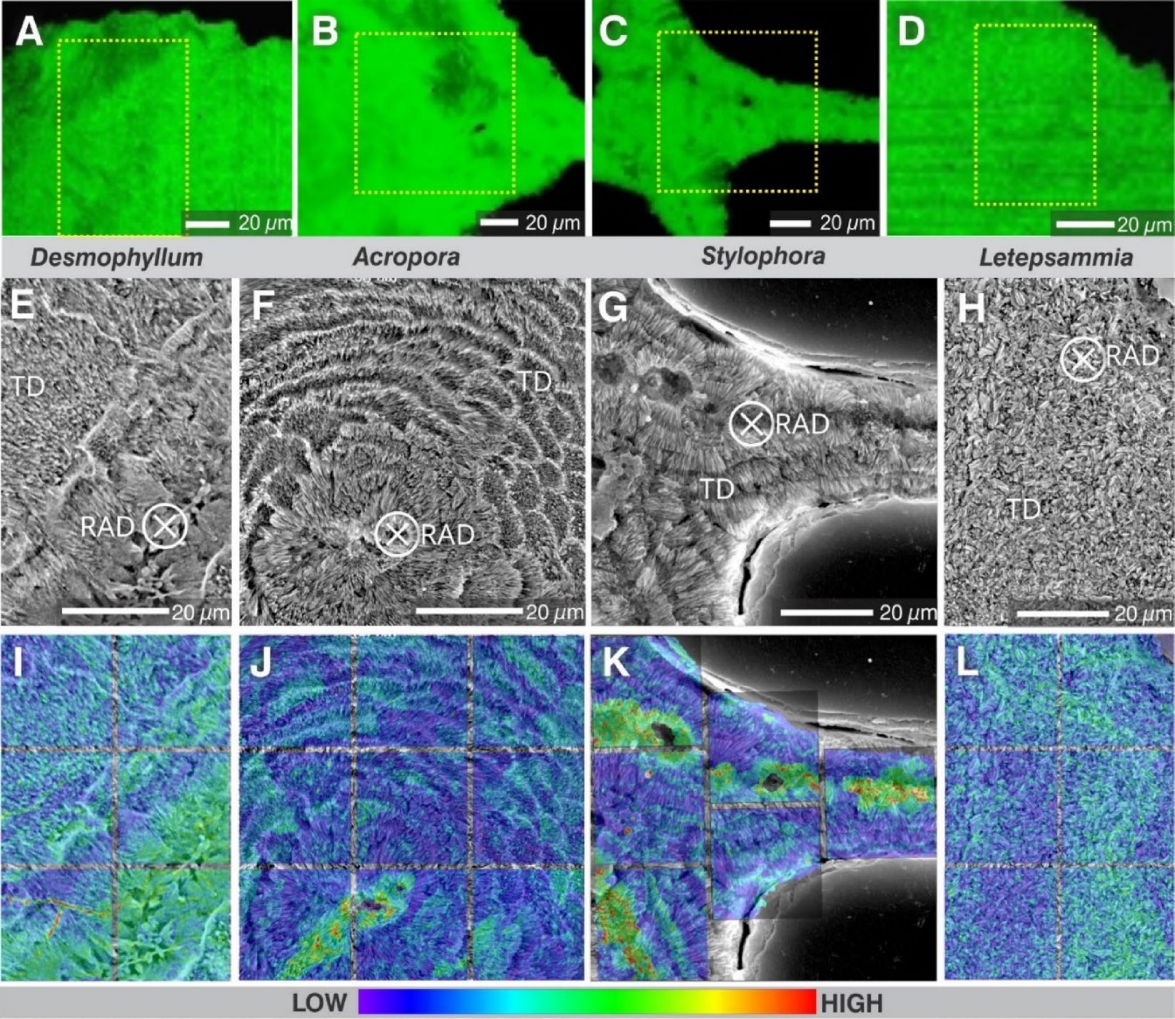
Coral skeletons subjected to isotopic exchange experiments after exposure to artificial seawater analogue (ASW) with a ^18^O/^16^O ratio of 0.30. (**A**–**D**), Raman maps showing aragonite as the only calcium carbonate phase detected in all experimentally treated samples (and in all controls). (**E**–**L**), Microstructural organization (RADs and TDs) of sectioned coral skeletons (**E**–**H**) with overlaid NanoSIMS maps (M–P) that show the relative oxygen isotope enrichments after exposure to ASW with an ^18^O/^16^O ratio of 0.30.

The skeletons were immersed for 7 days at 50 °C in artificial seawater analogue enriched in ^18^O (with an ^18^O/^16^O ratio of 0.30) and chemically in equilibrium with aragonite. After incubation experiments, all skeletal samples exhibited exclusively aragonitic mineralogy (Fig. 2A-D, Supplementary Fig. 1), i.e., no sign of aragonite-to-calcite transformation and no discernable differences in surface texture or skeletal microstructure compared to unaltered specimens (Fig. 1E-H). However, NanoSIMS mapping revealed that the ^18^O-enriched seawater analogue had penetrated and isotopically exchanged with the skeletons from all four species included in the study (Fig. 2I-L, Supplementary Fig. 2A-D). As expected, clear ^18^O**-**enrichments were observed in RAD regions (Supplementary Fig. 3A-D), which are generally considered most susceptible to diagenesis due to their nanogranular texture and high content of organic material (Supplementary Fig. 2E-H) . ^18^O**-**enrichment was also clearly observed in the TDs, particularly prominent within the fiber bundles in *Desmophyllum* (Supplementary Fig. 3E) and the shingles in *Acropora* (Supplementary Fig. 3F), with slightly lower enrichment and less distinct distribution pattern in *Stylophora* (Supplementary Fig. 3G) and, finally, a more diffusely distributed ^18^O**-**enrichment in the TDs of *Letepsammia* (Supplementary Fig. 3H). These ^18^O-enrichment distributions directly reflect the architecture of the TDs. Locally, the ^18^O-enriched areas, as revealed by NanoSIMS mapping, showed the following maximum values ^18^O enrichment expressed in arts-per-thousand relative to the measured ^18^O/^16^O ratio of a pristine skeletal fragment from each species): in *Desmophyllum* RADs were enriched by up to 3900‰ and TDs up to 2200‰; in *Acropora* the RADs exhibited enrichment up to 4000‰, whereas the shingle-like TDs showed enrichment up to 1500‰; in *Stylophora*, RADs were enriched by up to 5500‰ whereas TDs showed enrichment ranging from 1200‰-1500‰; in *Letepsammia* the ^18^O-enrichment was more homogeneously distributed, with maximum values between 1100‰ and 1800‰. This is consistent with bulk skeleton oxygen isotope measurements that, after incubation in seawater analogue in chemical equilibrium with aragonite and with δ^18^O_VSMOW_ = 1000‰, exhibited ^18^O-enrichments relative to pristine fragments of the coral of 3.23‰ in *Desmophyllum*, 3.06‰ in *Acropora*, 1.81‰ in *Stylophora* and 1.67‰ in *Letepsammia* (Table 1).

**Table 1:**
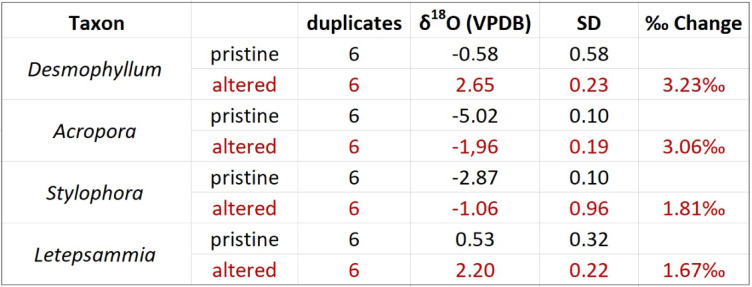
Bulk isotope δO^18^ measurements of original (pristine) and altered aragonite scleractinian coral samples. The samples were exposed for 7 days at 50 °C to ^18^O-enriched (δ^18^O_VSMOW_ = 1000‰) artificial seawater analogue saturated with respect to aragonite.

These experiments and the NanoSIMS isotope imaging clearly suggest that the microstructural organization of a coral skeleton is the primary feature guiding secondary modification of the oxygen isotope composition through exchange with pore water. The fine-scale organization of aragonitic biocrystals results from a precisely orchestrated and spatially delineated biomineralization process that consistently produces similar microstructures across distinct scleractinian coral clades^6,7,16,18^. The bulk of these skeletons have a microstructural architecture consisting of a three-dimensional organization of TDs fiber bundles with a corresponding distribution of organic components between/within these fibers. Consistent with observations in foraminiferal tests^8–11^in all the examined coral skeletons, regions enriched in organic matter act as ‘highways’ for water penetration and isotopic exchange. It can therefore be inferred that, when skeletons of different fossil coral species are exposed to pore water, isotope exchange will be heterogeneous and reflect these microstructures. This creates a range of susceptibility to oxygen isotope exchange with pore water that could mimic and hence be interpreted as part of the otherwise well documented ‘*vital effect*’, as samples of different coral species from the same location may (and do) exhibit differences in isotopic composition. However, bulk oxygen isotope signatures of fossil skeletons from the same location could in part result from different levels of isotope exchange influenced by the species-dependent microstructural organization of the skeleton, with less ultrastructural complexity (*Letepsammia*, *Stylophora*) conferring less susceptibility to O-isotope exchange than higher ultrastructural complexity (*Desmophyllum* and *Acropora*). This finding consequently introduces skeletal ultrastructure as a criterium when searching for and utilizing fossil aragonitic coral skeletons for paleoenvironmental reconstructions.

## Concluding remarks

A key objective of this study was to investigate if the composite organic-mineral architecture and complex nano- to microscale organization of coral skeletons result in different rates of isotopic exchange without phase transformation from aragonite to calcite. With experiments using moderately elevated temperatures and ^18^O-enriched pore waters we could visualize patterns of isotopic modification correlated with the ultrastructural diversity of modern scleractinian coral skeletons. The fact that such isotope exchange can take place without changes to the original mineralogy (i.e., no aragonite to calcite transformation) challenges the widespread assumption that preservation of aragonitic mineralogy and original skeletal microstructures in fossil coral skeletons ensures retention of their original geochemical signatures, with direct implications for the interpretation of isotopic records in paleoclimate reconstructions based on fossil corals.

The risk of significant bias in isotopic records from aragonitic fossils is generally considered low because, when aragonitic skeletons are exposed to pore waters capable of altering their isotopic composition, recrystallization of the metastable coral aragonite to calcite commonly occurs. However, instances are known of fossil coral skeletons (Late Jurassic) preserved in porous rock exposures thus subjected to penetrating meteoric and groundwater. These fossils have retained their original aragonitic mineralogy (probably stabilized by high concentrations of iron ions in the pore waters). Their oxygen isotope compositions^19^ are inconsistent with their colonial skeletal macro-morphologies that are typical of symbiotic taxa. We propose that the oxygen isotope compositions of such fossil coral skeletons might be heavily influenced by secondary isotopic exchange process through the mechanism identified in this study.

## Electronic supplementary material

Below is the link to the electronic supplementary material.

Supplementary Material 1

## Data Availability

Data generated or analysed during this study are included in this published article and its supplementary information files.

## References

[1] Gothmann, A. M. et al. Fossil corals as an archive of secular variations in seawater chemistry since the mesozoic. *Geochim. Cosmochim. Acta*. **160**, 188–208 (2015).

[2] DeLong, K. L., Quinn, T. M., Taylor, F. W., Lin, K. & Shen, C. C. Sea surface temperature variability in the Southwest tropical Pacific since AD 1649. *Nat. Clim. Chang.* **2**, 799–804 (2012).

[3] Corrège, T. et al. Interdecadal variation in the extent of South Pacific tropical waters during the younger Dryas event. *Nature* **428**, 927–929 (2004). 15118722 10.1038/nature02506

[4] Stolarski, J. Three-dimensional micro- and nanostructural characteristics of the scleractinian coral skeleton: A biocalcification proxy. *Acta Palaeontol. Polonica*. **48** (2003).

[5] Sorauf, J. E. & Cuif, J. P. Biomineralization and diagenesis in the scleractinia: part 2, diagenesis. *Bull. Tohoku Univ. Museum*. **1**, 152–163 (2001).

[6] Stolarski, J. et al. The ancient evolutionary origins of Scleractinia revealed by azooxanthellate corals. *BMC Evol. Biol.* **11**, 316 (2011). 22034946 10.1186/1471-2148-11-316 PMC3224782

[7] Janiszewska, K. et al. A unique skeletal microstructure of the deep-sea micrabaciid scleractinian corals. *J. Morphol.* **272**, 191–203 (2011). 21210490 10.1002/jmor.10906

[8] Chanda, P., Gorski, C. A., Oakes, R. L. & Fantle, M. S. Low temperature stable mineral recrystallization of foraminiferal tests and implications for the fidelity of geochemical proxies. *Earth Planet. Sci. Lett.* **506**, 428–440 (2019).

[9] Cisneros-Lazaro, D. et al. Fast and pervasive diagenetic isotope exchange in foraminifera tests is species-dependent. *Nat. Commun.* **13**, 113 (2022). 35013292 10.1038/s41467-021-27782-8 PMC8748890

[10] Cisneros-Lazaro, D. et al. Fossil biocalcite remains open to isotopic exchange with seawater for tens of millions of years. *Sci. Rep.* **14**, 24933 (2024). 39438650 10.1038/s41598-024-75588-7 PMC11496820

[11] Adams, A. et al. Rapid grain boundary diffusion in foraminifera tests biases paleotemperature records. *Commun. Earth Environ.* **4**, 144 (2023). 38665181 10.1038/s43247-023-00798-2 PMC11041775

[12] Weiner, S., Levi-Kalisman, Y., Raz, S. & Addadi, L. Biologically formed amorphous calcium carbonate. *Connect. Tissue Res.* **44**, 214–218 (2003). 12952200

[13] Stolarski, J. & Mazur, M. Nanostructure of biogenic versus abiogenic calcium carbonate crystals. *Acta Palaeontol. Pol.* **50**, 847–865 (2005).

[14] Cuif, J. P., Dauphin, Y. & Sorauf, J. E. *Biominerals and Fossils Through time* (Cambridge University Press, 2010).

[15] Gilbert, P. U. P. A. et al. Biomineralization: integrating mechanism and evolutionary history. *Sci. Adv.* **8**, eabl9653 (2022). 35263127 10.1126/sciadv.abl9653 PMC8906573

[16] Stolarski, J. et al. A unique coral biomineralization pattern has resisted 40 million years of major ocean chemistry change. *Sci. Rep.* **6**, 27579 (2016). 27302371 10.1038/srep27579 PMC4908604

[17] Kitahara, M. V., Fukami, H., Benzoni, F. & Huang, D. The new systematics of scleractinia: integrating molecular and morphological evidence. In *The Cnidaria, Past, Present and Future: The world of Medusa and her sisters* (eds. Goffredo, S. & Dubinsky, Z.) 41–59 (Springer International Publishing, 2016).

[18] Seiblitz, I. G. L. et al. Caryophylliids (Anthozoa, Scleractinia) and mitochondrial gene order: insights from mitochondrial and nuclear phylogenomics. *Mol. Phylogenet Evol.* **175**, 107565 (2022). 35787457 10.1016/j.ympev.2022.107565

[19] Gruszczyński, M., Hoffman, A., Małkowski, K., Tatur, A. & Hałas, S. Some geochemical aspects of life and burial environments of late jurassic scleractinian corals from Northern Poland. *Neues Jahrb Geol. Paläontol*. **11**, 673–686 (1990).

