# Supplementary material for: Rapid isotopic exchange in mineralogically unaltered coral skeletons
Source: Sci Rep. 2025 Jul 1;15:20986. doi: 10.1038/s41598-025-06327-9 (PMC12216655; doi:10.1038/s41598-025-06327-9)
Supplement: Supplementary file 1 — Supplementary Material 1 [file 41598_2025_6327_MOESM1_ESM.docx]

Supplementary Information for:

Rapid isotopic exchange in mineralogically unaltered coral skeletons

**Jarosław Stolarski^1*^, Deyanira Cisneros-Lazaro^2^, Arthur Adams^2^, Katarzyna Janiszewska^1^, Anders Meibom^2,3^**

*^1^Institute of Paleobiology, Polish Academy of Sciences, PL-00-818 Warsaw, Poland;*

*^2^Laboratory for Biological Geochemistry, School of Architecture, Civil and Environmental engineering, Ecole Polytechnique Fédérale de Lausanne (EPFL), Lausanne, CH-1015 Switzerland;*

*^3^Center for Advanced Surface Analysis, Institute of Earth Science, University of Lausanne, CH-1015 Lausanne, Switzerland.*

This file includes:

SI Methods

SI Figures: 1-4

SI References

**SI Methods**

**Isotopic exchange experiments**. The coral skeletons were treated with a 5% NaOCl solution for 1 hour at room temperature to remove any residual organic matter, then rinsed in Milli-Q^®^ water and placed in an oven to dry at 50 °C for 1 day. Following desiccation, pieces of coral skeleton (septa) approximately 5 mm in length were broken into smaller pieces such that they could fit into the gold capsules which measure 5 mm in diameter. For bulk measurements, the capsules were filled with ~40 mg of the ^18^O-enriched seawater analogue with δ^18^O_VSMOW_ = 1000 ‰ and saturated with respect with aragonite (SI=1), whereas for NanoSIMS measurements, the ^18^O/^16^O ratio was 0.30 (which corresponds to a δ^18^O_VSMOW_ of 212’730 ‰). The sealed capsules were in both cases placed in an oven at 50 °C for seven days. After the reaction, the gold capsules were quenched in cool water for less than a minute, cleaned with Milli-Q^®^ water and ethanol, then desiccated at 50 °C in an oven followed by 24 hours of vacuum desiccation at room temperature.

**Raman analyses and mapping**. The measurements were performed with a Renishaw inVia Qontor confocal micro-spectrometer in back-scattered geometry (Institute of Earth Sciences, University of Lausanne) using a 785 nm Nd laser, a 1024 x 256 pixel CCD detector, a Rayleigh rejection edge filter, and a Leica optical microscope with a motorized XYZ stage. Daily calibrations were conducted with a silicon standard (520.5 cm^-1^ peak). A grating of 1200 grooves/mm was used for the analyses. These analytical conditions yielded a spectral resolution better than 1 cm^-1^. Raman maps for each coral sample consisted of between 4600 and 7200 analyses, with each analysis lasting 1 second. The WiRE 5.5 software was used to deconvolute the data. An untreated coral specimen was used as the aragonite standard and the prismatic calcitic layer of a *Pinna nobilis* bivalve was used as the calcite standard for generating maps using a non-negative least squares (NNLS) fitting algorithm commonly used for mineral detection using Raman[^1^](https://paperpile.com/c/WMMWzG/HAWN). Raman mapping of multiple, spatially distributed areas across all samples showed that aragonite was the only calcium carbonate phase detected in experimentally treated and control samples.

**Bulk isotope analyses**. Coral oxygen isotope compositions were measured by phosphoric acid digestion at 70 °C at the University of Lausanne on a Finnigan Delta V Advantage mass spectrometer coupled to a GasBench II [^2^](https://paperpile.com/c/WMMWzG/PIsp). Coral fragments were crushed and 6 aliquots of 150 μg of coral were analyzed for each genus. Isotope ratios were corrected online for phosphoric acid fractionation by a Carrara marble internal standard^2^. Analytical precision of the isotope analysis was approximately ±0.1‰ based on replicate standard analyses.

**NanoSIMS analysis**. Dried and vacuum desiccated coral samples were embedded in epoxy (EpoThin2, Struers) in aluminum rings, vacuum pumped to remove air bubbles and hardened overnight. Sample surfaces were then polished using increasingly finer grained diamond paste (from 15 to 0.25 µm) and coated with ca. 15 nm Au. NanoSIMS imaging of the resulting distribution of ^18^O-enrichment in the coral skeleton was carried out with a 16 keV Cs^+^ primary ion beam focused to a spot-size of about 120 nm (ca. 0.7 pA on the sample surface). Positive charge build-up on the surface was compensated with an electron gun. The multi-collector system simultaneously counted the following ions in individual electron-multiplier detectors with a mass resolving power of ~9000 (Cameca definition): ^16^O^-^, ^18^O^-^, ^14^N^12^C^-^. ^31^P^-^, ^32^S^-^, ^16^O^24^Mg^-^, and ^16^O^40^Ca^-^. Areas of 25×25 µm^2^ were imaged with a raster of 256×256 pixels and a dwell-time of 5 milliseconds per pixel. Up to 25 sequential images were produced for each area and were accumulated and drift corrected using L'image (developed by Dr. Larry Nittler, Carnegie Institution of Washington, USA). Oxygen isotope compositions were reported as δ^18^O (in parts-per-thousand) relative to unaltered reference coral skeletons prepared and analyzed in an identical manner. To determine the areas for NanoSIMS analysis, the polished samples were briefly surficially etched using a diluted form of Mutvei’s solution (2 vol % glutaraldehyde + 0.1 vol % acetic acid). This mild etching allowed for the visualization of RAD and TD microstructures, aiding in the selection of representative regions. Following etching and microstructural assessment, the samples were re-polished to expose a fresh, unaltered surface for subsequent NanoSIMS analysis.

**Scanning Electron Microscopy (SEM) imaging**. Polished sections analyzed with NanoSIMS were then lightly etched in Mutvei’s solution following described procedures^3^, and then rinsed with Milli-Q water and air-dried. After drying, the specimens were put on stubs with double-sticking tape and sputter-coated with conductive platinum film. Analyses were made using a Phillips XL20 scanning electron microscope at the Institute of Paleobiology, Warsaw, Poland.

**Supplementary Figures**


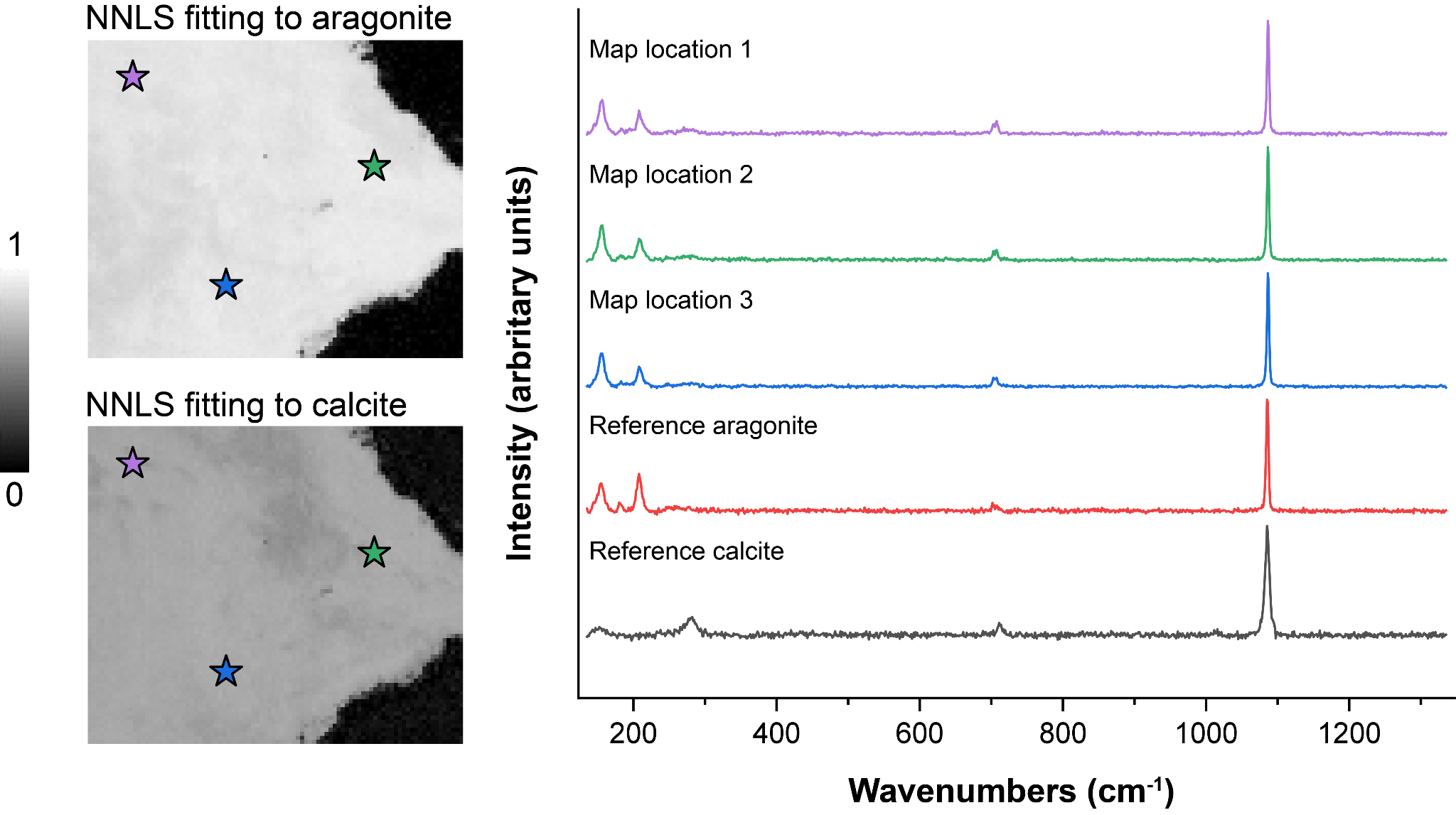


**SI Figure 1**. **Raman maps of *Stylophora* sectioned skeleton, after exposure to artificial seawater with an ^18^O/^16^O ratio of 0.30, and point spectra of 3 locations as well as reference calcite and aragonite spectra**. Non-negative least squares (NNLS) mapping fitting to aragonite and calcite shows output values consistent only with aragonite (both before and after experiments).


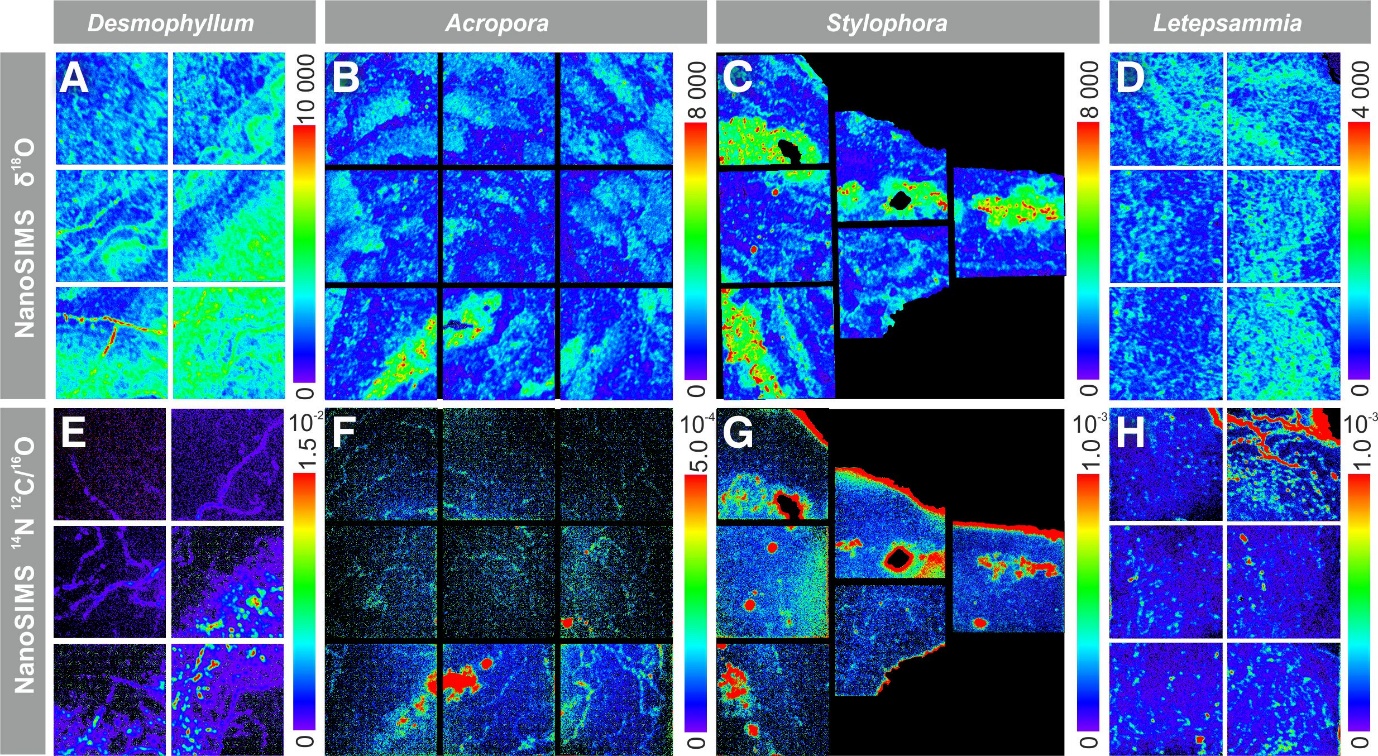


**SI Figure 2**. **NanoSIMS images of the polished skeletons of four scleractinian corals after exposure to the seawater analogue with an ^18^O/^16^O ratio of 0.30.** *Desmophyllum* (**A**, **E**), *Acropora* (**B**, **F**), *Stylophora* (**C**, **G**), *Letepsammia* (**D**, **H**). Direct comparison between NanoSIMS maps of relative oxygen isotope enrichment of structural regions (A-D), and NanoSIMS isotope ratio maps with ^14^N serving as a proxy for the presence of intra-skeletal organic compounds (E-H); note that enrichment of ^14^N at the edge of samples (G, H) most likely corresponds to occurrence of some organic remnants on the surface of the sample.


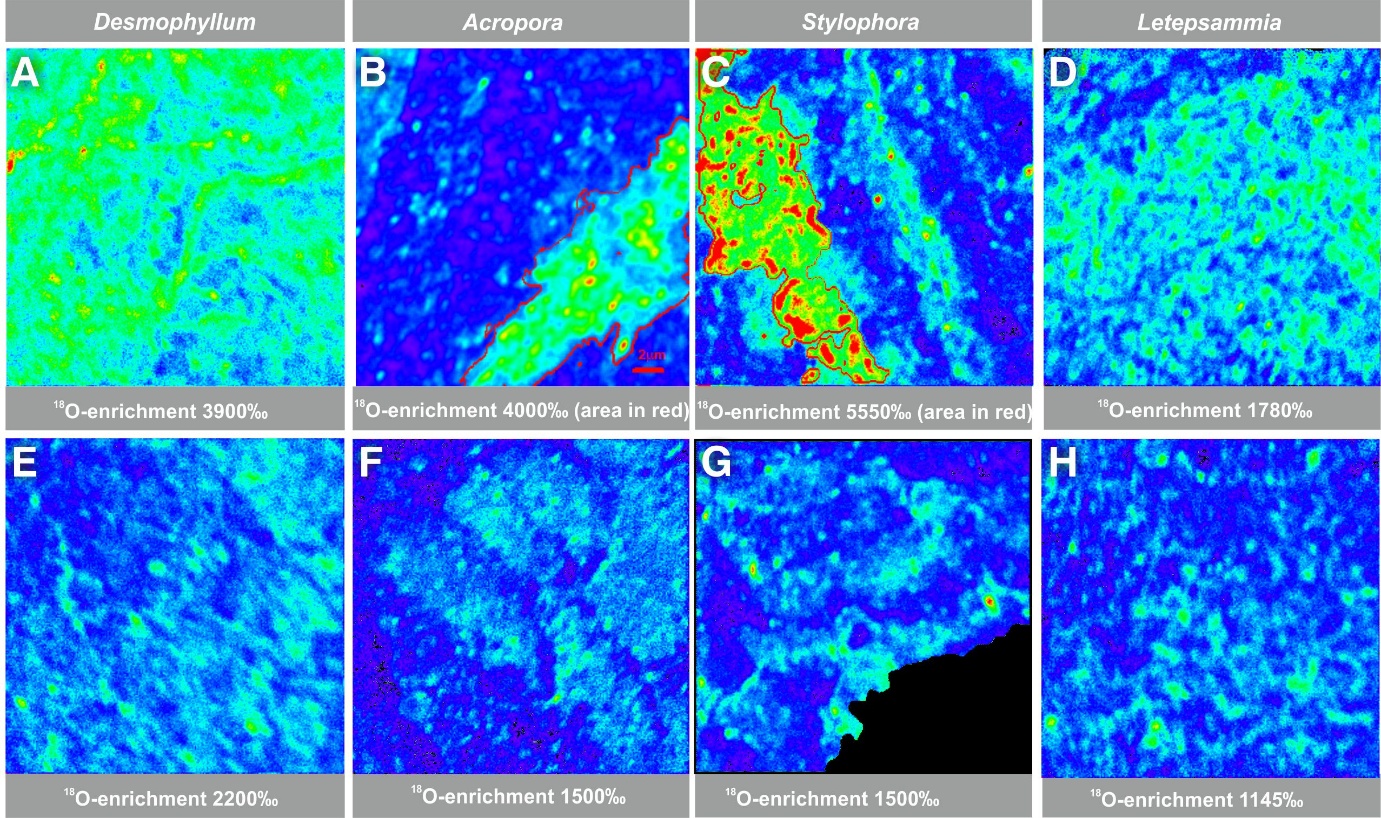


**SI Figure 3**. **NanoSIMS O-isotope maps in RAD and TD regions of the polished skeletons of four scleractinian corals after exposure to the seawater analogue with an ^18^O/^16^O ratio of 0.30.** RADs **(A-D)** showed higher values of ^18^O-enrichment in comparison to TDs (**E-H**), which were also significantly ^18^O-enriched (enrichment expressed as δ^18^O in parts-per-thousand relative to the measured ^18^O/^16^O ratio of a pristine skeletal fragment from each species): *Desmophyllum* (**A, E**), *Acropora* (**B**, **F**), *Stylophora* (**C**, **G**), *Letepsammia* (**D**, **H**).

**SI References**

1. [Mark, H. & Workman, J., Jr. *Chemometrics in Spectroscopy*. (Academic Press, 2018).](http://paperpile.com/b/WMMWzG/HAWN)

2. [Spötl, C. & Vennemann, T. W. Continuous-flow isotope ratio mass spectrometric analysis of carbonate minerals. *Rapid Commun Mass Spectrom* **17**, 1004–1006 (2003).](http://paperpile.com/b/WMMWzG/PIsp)

3. [Schöne, B. R., Dunca, E., Fiebig, J. & Pfeiffer, M. Mutvei’s solution: An ideal agent for resolving microgrowth structures of biogenic carbonates. *Palaeogeogr. Palaeoclimatol. Palaeoecol.* **228**, 149–166 (2005).](http://paperpile.com/b/WMMWzG/eJGV)
